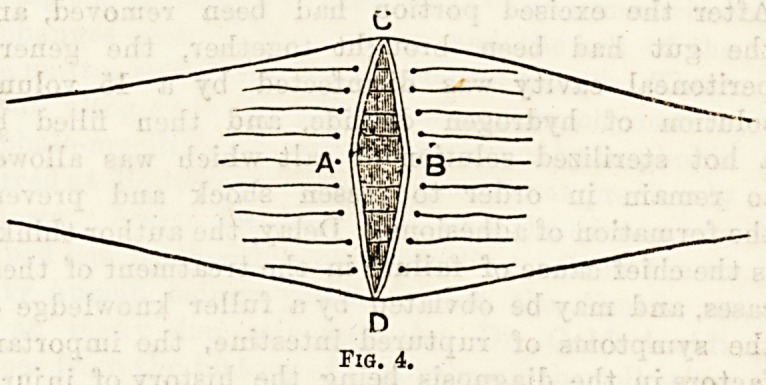# Progress in Surgery

**Published:** 1894-11-17

**Authors:** 


### Progress in Surgery.

### SURGERY OF THE INTESTINES.

*(Continued from page 101.)*

**Intestinal Perforation.**—Cordier reports[9] a case which was due to the tension of a band across the ileum, which eleven days before had caused symptoms of acute obstruction. The rent in the bowel was sutured, the portion of gut which was strangulated by the band was released, and lateral anastomosis, by means of Murphy's button, was done around the seat of constriction. The operation lasted thirty minutes. The patient recovered, but the button had not been passed four weeks after operation. Dr. Cayley and Mr. Bland Sutton relate[10] a case of perforation of the bowel in Typhoid Fever about the twenty-fourth day. On withdrawing a coil of intestine from the pelvis a perforation was found in the centre of an oval ulcer, the outline of which was plainly apparent through the intestinal wall. The ulcer was excised by an oval incision, the cut edges of the mucous membrane drawn together by continuous suture, and then the serous surfaces were brought into apposition by eleven Lembert's sutures. The operation lasted nearly an hour. The patient lived till the sixth day. Post-mortem examination showed that a stitch had sloughed exactly in the middle of the line of suture. Mr. Sutton thought it might in future be better not to excise and suture the ulcer, but, after washing out the peritoneal cavity, to attach the perforated bowel to the abdominal incision, and leave a fistula which could be dealt with subsequently.

**Enterectomy for Rupture of the Ileum.**—Wiggin reports[11] a case of contusion and rupture of the ileum without external wound, which was successfully treated by primary enterectomy and circular enterorraphy after Maunsell's method. The patient, a boy of fifteen, had been kicked by a horse in the right lumbar region, and this was followed by symptoms of traumatic peritonitis. Laparotomy was performed about thirty hours after the injury. After the excised portion had been removed, and the gut had been brought together, the general peritoneal cavity was disinfected by a 15 volume solution of hydrogen dioxide, and then filled by a hot sterilized solution of salt which was allowed to remain in order to lessen shock and prevent the formation of adhesions. Delay, the author thinks, is the chief cause of failure in the treatment of these cases, and may be obviated by a fuller knowledge of the symptoms of ruptured intestine, the important factors in the diagnosis being the history of injury, persistent nausea, hæmorrhage from the bowels, prolonged shock, rise of temperature, increased frequency of respiration, rigidity of the abdominal muscles, persistent pain with or without pressure, and the facial expression. W. Thelwall Thomas also narrates[12] a successful case of enterorraphy for ruptured intestine. The operation was performed about twenty-four hours after the accident which caused the rupture of the gut. Mr. Battle reports[13] an unsuccessful case of enterectomy for rupture of the gut due to the kick of a horse. The operation was performed about five-and-a-half hours after the accident. The end to end approximation of the intestines, however, gave way on the fifth day after operation, and the patient died on the following day in spite of the formation of an artificial anus. Post-mortem: the opening was found to be only twenty inches from the pylorus. The hope of success in these cases is in early intervention. Of fifteen cases recorded since 1890 there had been seven recoveries.

**Perforating Ulcer of Duodenum.**—Mr. Percy Dean records[14] a case which occurred in a female patient, aged thirty-two. On admission it was stated that she had had pains in the epigastrium for a fortnight. with sickness and constipation for eight days. Twenty-four hours before admission she became much worse, with severe pains in the pit of the stomach. She was thought to be suffering from acute general peritonitis, due to mechanical obstruction, but as on opening the abdomen no such cause was found to account for the peritonitis a further search was made, and an ulcer was discovered on the anterior surface of the duodenum, about three-quarters of an inch below the pylorus. This part of the duodenum was excised with scissors, and the resulting aperture sutured. She made a good recovery, and was up and about on the thirtieth day. Three days before she was discharged, however, she developed symptoms of intestinal obstruction. This was found, on opening the abdomen, to be due to a band. This was released, but the patient died thirty-six hours later. Mr. Dean concludes, therefore, that if after abdominal section an attack of acute abdominal pain and intestinal obstruction supervene, an exploratory operation should be performed as soon as possible.

**Jejunostomy.**—In order to utilise the pancreatic and hepatic secretions, in cases where it is proposed to feed through an artificial fistula, Maydl, after dividing the loop of jejunum transversely, drew out the distal end still farther, and made another opening lower down, into which the proximal end of the gut was sutured. The tranverse wound of the distal end was then sewed into the abdominal wound, through which food could be introduced. Albert reports[15] a modification of this method which he thinks is simpler. A loop of jejunum was drawn out. The gut was not cut across, but anastomosis was made at the base of the loop between the distal and proximal sections. Parallel to the first abdominal wound and four centimetres above it a second incision two centimetres long was made through the skin. Between these two wounds the skin was undermined so that a bridge of skin was formed. The loop of jejunum was then dragged under this bridge and sutured to the edges of the upper wound. The skin was sutured entirely over the lower wound, leaving the gut with a skin covering. The anastomosis lay directly behind the peritoneal wound in the abdominal cavity. On the fourth day after the operation the intestine was opened with the cautery. He reports[16] two cases, both unsuccessful. E. Hahn observes[17] that in the operation of jejunostomy the fistula must be made high up in the small intestine, otherwise death will occur from inanition. The indications for the operation are (1) corrosion of the stomach and œsophagus when a fatal result is to be feared; (2) carcinoma involving the cardia and lower end of the œsophagus when gastrostomy is impossible; and (3) carcinoma of the pylorus where no other operation is possible.

**Circular Enterorraphy** by means of a decalcified bone tube of the shape here given has been the subjects of experiment[18] on seven dogs by R. Cozens Bailey. For use in the human subject the most convenient length is two inches, the diameter varying from half an inch to one and one-eighth inches, according to the size of the gut. The tube is thus used.

Fig. 1.—Decalcified Bone Tube.

After the portion of intestine has been resected a stitch is passed through all the coats of the bowel and the mesentery of one side, and then on through mesentery and bowel of the other side and tied, thus bringing the divided ends together and preventing retraction of the mesentery. The tube is then inserted and the two ends slipped together evenly over it; each is in turn brought down well over its corresponding groove in the tube and firmly secured by a circular ligature passed through the gap in the mesentery.

Fig. 2.—Operation complete.

Any excess of tissue beyond the ligatures is removed with scissors, and the operation is completed by bringing the serous surfaces together with Lembert's sutures, five in number, one on each side of the mesenteric attachment, the remainder round the rest of the circumference. Of the seven dogs thus operated upon one died on the fifth day from peritonitis, one was killed on the sixth day, one died on the twenty-seventh day from obstruction, one was killed on the thirtieth day, one on the forty-fourth day, one on the sixty-fifth day, and one on the eighty-seventh day. In all the immediate results were good, there being no escape of fæces; and in the five which lived for periods ranging from one to three months the condition of the scar was satisfactory.

**Sarcoma of the Meso-Sigmoid.**—A case of this rare (when the growth is not secondary) condition is reported[19] by Mr. W. Arbuthnot Lane. He performed abdominal section, and removed the growth with the mesentery about it and the corresponding portion of bowel, and united the two ends of the gut by means of Murphy's button. In consequence of the large size of the glandulæ epiploicæ there was some difficulty in obtaining proper coaptation of the two parts of the divided intestine, and feeling that the presence of such a large quantity of fat exposed the patient to the risk of imperfect union, he wrapped the buttoned intestine in gauze and replaced it in the peritoneal cavity. Five days after the gauze was removed and fresh material introduced. The patient recovered, leaving the hospital with a small fistulous track which discharged a little pus. Mr. Lane thinks the gauze tampon secures the patient from immediate risk; but he asks if it be possible that matting of intestines around the tamponaded area may, at a subsequent period, produce risks of intestinal obstruction.

**Large Pseudo-Diverticulum of the Duodenum** existed in a case operated upon by L. S. Pilcher.[20] The cavity, which contained a puriform fluid with pieces of partly-digested vegetable material, was drained, but the patient died on the third day from peritonitis. Dr. Pilcher thinks that the most plausible theory of the formation of these pouches is that which presupposes the occurrence of thrombosis of one of the duodenal arterioles, necrosis and digestion of the portion of the duodenal wall whose nutrition was thus enfeebled or destroyed, gradual invasion of the post peritoneal connective tissue opened into by this process of ulceration forming a sac with walls more or less thick, formed largely of inflammatory connective tissue.

**Enteroplasty** has been performed in two cases[21] by H. W. Allingham. The operation which he proposes for innocent strictures of the intestines is a simple one, and similar in its principles to the operation of pyloroplasty, consisting in a longitudinal division of the strictured intestine and a transverse approximation of the cut edges. The steps of the operation are sufficiently well seen by means of the accompanying diagrams.

Fig. 3.

Fig. 4.

**Intussusception.**—The treatment of this condition by early laparotomy seems[22] to be gaining favour. Dr. Ainsley and Dr. Beatley and Mr. Ridley[23] report successful cases. The former case was operated upon six and a half hours after the commencement of the symptoms—the latter case, forty-one hours after. Inflation, and injection of water had been previously tried in the respective cases.

[9] Journ. of the Amer. Med. Assoc., vol. xxii., No. 6, and Therapeutic Gazette, March 15, 1894. [10] Lancet, March 17, 1894. [11] N. Y. Med. Journ., Jan. 20, 1894, and Brit. Med. Journ., May 5, 1894. [12] Brit. Med. Journ., June 23, 1894, p. 1,355. [13] Brit. Med. Journ., May 5, 1894, p. 968. [14] Medical Week, May 11, 1894, and Brit. Med. Journ. May 12, 1894. [15] Annals of Surgery, August 1894, p. 239. [16] Wein. Med. Woch, No. 2. 1894. [17] Deut. Med. Woch, July 5, 1894, and Brit. Med. Journ., August 4, 1894. [18] Brit. Med. Journ., July 14, 1894, p. 65. [19] Lancet, April 21, 1894. [20] Annals of Surgery, July, 1894 p. 62. [21] Lancet, June 23, 1894, p. 1,551. [22] Lancet, May 19, 1894, p. 1,247. [23] Brit. Med. Journ., April 28, 1894.

## Figures and Tables

**Fig. 1. f1:**
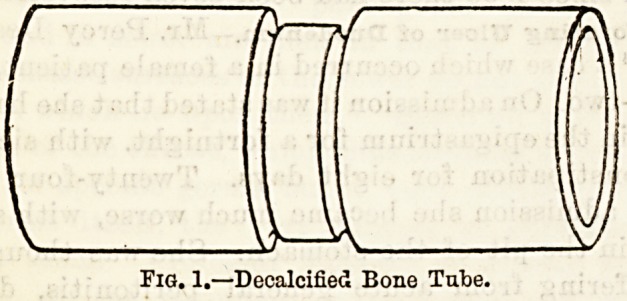


**Fig. 2. f2:**
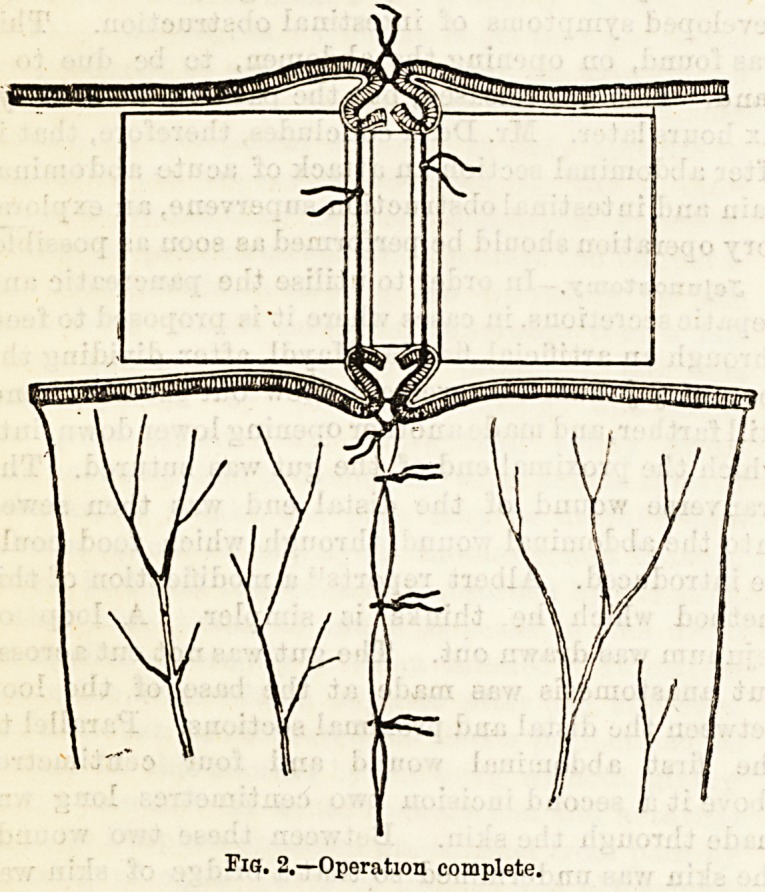


**Fig. 3. f3:**
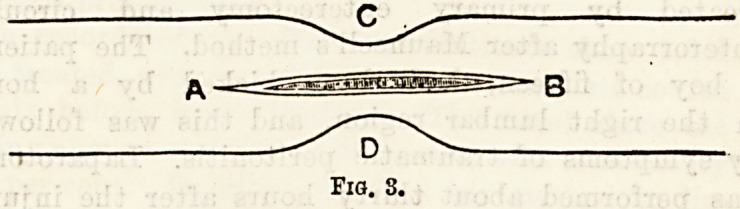


**Fig. 4. f4:**